# An Apoptosis-Associated Mammary Protein Deficiency Leads to Enhanced Production of IgM Antibodies against Multiple Damage-Associated Molecules

**DOI:** 10.1371/journal.pone.0068468

**Published:** 2013-07-12

**Authors:** Miho Chikazawa, Natsuki Otaki, Takahiro Shibata, Takehiko Yasueda, Tsukasa Matsuda, Koji Uchida

**Affiliations:** 1 Laboratory of Food and Biodynamics, Graduate School of Bioagricultural Sciences, Nagoya University, Nagoya, Japan; 2 Laboratory of Molecular Bioregulation, Graduate School of Bioagricultural Sciences, Nagoya University, Nagoya, Japan; Case Western Reserve University, United States of America

## Abstract

Milk fat globule epidermal growth factor 8 (MFG-E8) is a protein that binds to apoptotic cells by recognizing phosphatidylserine and enhances the engulfment of apoptotic cells by macrophages. Many apoptotic cells are left unengulfed in the germinal centers of the spleen in the MFG-E8-deficient (MFG-E8^−/−^) mice, and these mice develop an autoimmune disease resembling human systemic lupus erythematosus. We found that the MFG-E8 deficiency was accompanied by the increased production of immunoglobulins. Further Western blot and ELISA analyses validated the increase in the IgM levels in the MFG-E8^−/−^ mice. It was also revealed that the sera from the MFG-E8^−/−^ mice cross-reacted with oxidation-specific epitopes generated upon incubation of serum albumin with the peroxidized lipids. Among the modified proteins with several unsaturated aldehydes of chain lengths varying from three to nine carbons, the MFG-E8^−/−^ mice sera exclusively cross-reacted with the protein-bound 4-oxo-2-nonenal (ONE), a highly reactive aldehyde originating from the peroxidation of ω6 polyunsaturated fatty acids. In addition, the IgM monoclonal antibodies (mAbs) that selectively cross-reacted with the ONE-modified proteins were generated from the MFG-E8^−/−^ mice. A subset of the ONE-specific IgM mAbs significantly recognized the late apoptotic and necrotic cells and enhanced the phagocytosis by macrophages. These data demonstrate that the impairment of the phagocytic clearance of apoptotic cells through MFG-E8 can lead to the generation of natural antibodies, which may play a critical role in removing multiple damage-associated molecules, including oxidation-specific epitopes and late apoptotic/necrotic cells.

## Introduction

**Figure 1:**
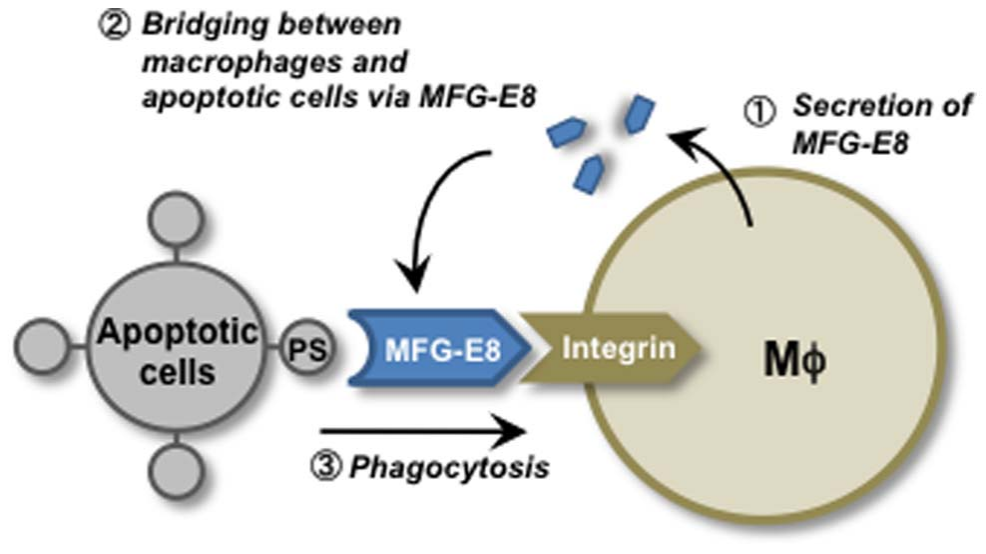
Engulfment of apoptotic cells via MFG-E8. MFG-E8, secreted by activated macrophages and immature dendritic cells (*first step*), binds to apoptotic cells by recognizing phosphatidylserine (PS) (*second step*) which enhances the engulfment of apoptotic cells by macrophages (*third step*).

Milk fat globule epidermal growth factor factor 8 (MFG-E8), originally found associated with milk fat globules in mammary glands, is a secreted protein present on a subset of phagocytes that actively engulf apoptotic cells [1]. It is expressed by macrophages and immature dendritic cells, including tingible-body macrophages and follicular dendritic cells at the germinal centers in the spleen and lymph nodes, thioglycollate-elicited peritoneal macrophages, granulocyte-macrophage colony stimulating factor-induced bone marrow-derived immature dendritic cells, and Langerhans cells in the skin [2]–[4]. MFG-E8 is also released by apoptotic endothelial cells in a caspase-3-dependent manner [5]. MFG-E8 contains one (human) or two (mouse) epidermal growth factor (EGF) domains in its N-terminal half, with the human and second mouse EGF domain carrying an RGD (Arg-Gly-Asp) motif. It has two factor-VIII-homologous domains (C1 and C2) in its C-terminal region. MFG-E8 associates with the αvβ3 or αvβ5 integrin on phagocytes via its RGD motif [6], binds tightly to phosphatidylserine through its C1 and C2 domains, and stimulates the engulfment of apoptotic cells (**Figure 1**) [1].

MFG-E8-deficient female (MFG-E8^−/−^) mice, particularly of the B6/129-mixed background, develop an age-dependent systemic lupus erythematosus (SLE)-type of autoimmune disease [2]. These mice produce high concentrations of anti-double-stranded DNA (dsDNA) and anti-nuclear antibodies and suffer from glomerular nephritis. When MFG-E8^−/−^ mice are immunized with keyhole limpet hemocyanin (KLH) to activate the B lymphocytes, many apoptotic cells are left unengulfed on the tingible-body macrophages in the germinal centers, confirming that MFG-E8 has a nonredundant role in vivo in the engulfment of apoptotic cells by the tingible-body macrophages. It is likely that the unengulfed dead cells in the MFG-E8^−/−^ mice undergo a secondary necrosis and release cellular components that activate the immune system to produce autoantibodies. Like Fas-deficient lpr mice, in which autoreactive B cells are activated by a T cell-independent, but Toll-like receptor- and B cell receptor-dependent mechanism [7], the released cellular components may activate autoreactive B cells in a BCR- and TLR-dependent manner. This activation of autoreactive B cells may be further enhanced by cytokines produced by macrophages in response to stimulation by the necrotic cells. A recent study by Peng and Elkon [8] has also shown that MFG-E8 controls the phagocytic ingestion of cell fragments as well as their intracellular processing into MHC-antigen complexes. In any case, since human patients with SLE often have a defect in the engulfment of apoptotic cells by the tingible body macrophages in the germinal centers [9], the MFG-E8-deficient mice provide a good model system for studying the molecular mechanisms by which endogenous cellular components extracellularly activate the immune system.

There is increasing evidence that lipid peroxidation is associated with autoimmune diseases, such as SLE. (i) SLE patients have an enhanced urinary excretion of isoprostanes, the well-established biomarkers of lipid peroxidation [10], (ii) the levels of the lipid peroxidation-derived short-chain aldehydes are significantly elevated in children with a high disease activity of SLE [11], and (iii) there are elevated levels of the oxidized low-density lipoproteins (LDL) together with elevated levels of antibodies (Abs) related to the oxidized LDL in female patients with SLE [12]. The involvement of lipid peroxidation was also suggested by the observation that the lipid peroxidation-derived epitopes were detected in the sera from the SLE patients and from the SLE-prone mice [13], [14]. These findings raised the possibility that the enhanced lipid peroxidation may be involved in the pathogenesis of autoimmune disorders. In the present study, we analyzed the MFG-E8^+/+^ and MFG-E8^−/−^ mice sera and found that the production of IgM was highly enhanced in the MFG-E8-deficient mice. In addition, we revealed that the IgM antibodies (Abs) present in high levels in the MFG-E8^−/−^ mice, specifically recognized the modified protein with 4-oxo-2-nonenal (ONE), a highly reactive aldehyde originating from the peroxidation of ω6 polyunsaturated fatty acids. Following the identification of ONE as the major source of epitopes, we generated several ONE-specific IgM monoclonal antibodies (mAbs) from the MFG-E8^−/−^ mice and characterized their specificity toward lipid peroxidation-derived protein ligands. Furthermore, we examined the ability of the ONE-specific IgM mAbs to bind to apoptotic and necrotic cells.

## Materials and Methods

### Materials

Calf thymus DNA was purchased from Sigma. Linoleic acid was obtained from Nu-Chek Prep, Inc. (Elysian, MN). Bovine serum albumin (BSA), crotonaldehyde, 2-pentenal, 2-hexenal, 2-heptenal, 2-octenal, 2-nonenal, and 2-decenal were obtained from Wako Pure Chemical Industries, Ltd. (Osaka, Japan). 13-hydroperoxy-9Z,11E-octadecadienoic acid (13-HPODE) was synthesized by a previously described method [15]. Acrolein was obtained from Tokyo Kasei (Tokyo, Japan). The sodium salt of malondialdehyde (MDA) was prepared by hydrolysis of malondialdehyde bis (diethyl acetal) [16]. The horseradish peroxidase (HRP)-linked anti-mouse IgA, anti-mouse IgD, anti-mouse IgG, anti-mouse IgM immunoglobulins (for immunoblot analysis), HRP-NeutrAvidin, and ECL (enhanced chemiluminescence) Western blotting detection reagents were obtained from GE Healthcare. The HRP-linked anti-mouse IgG and IgM immunoglobulins (for ELISA analysis) were purchased from Cappel Laboratories.

### Animals

MFG-E8^−/−^ mice were kindly provided by Dr. Shigekazu Nagata of Kyoto University [2]. All animal protocols were approved by the Animal Experiment Committee in the Graduate School of Bioagricultural Sciences, Nagoya University.

### Preparation of Lipid Peroxidation-modified Proteins

Modification of the protein by the lipid peroxidation products was performed by incubating BSA (1.0 mg/ml) with linoleic acid or arachidonic acid (5.0 mM), FeSO_4_(NH_4_)_2_SO_4_^.^6H_2_O (0.5 mM), and vitamin C (2.0 mM) in 0.1 M HEPES buffer (pH 7.4) with 10% ethanol at 37°C under atmospheric oxygen. After 2, 4, 8, 12, 24, 48 and 72 h, aliquots were collected and the reaction terminated by adding butylated hydroxyltoluene (1.0 mM) and diethylene triamine pentaacetic acid (100 µM).

### ELISA

A 100-µl aliquot of the antigen solution was added to each well of a 96-well microtiter plate and incubated for 20 h at 4°C. The antigen solution was then removed, and the plate was washed with phosphate-buffered saline (PBS) containing 0.5% Tween 20 (PBS/Tween). Each well was incubated with 200 µl of 4% Blockace (Yukijirushi, Sapporo, Japan) in PBS/Tween for 60 min at 37°C to block the unsaturated plastic surface. The plate was then washed three times with PBS/Tween. A 100-µl aliquot of a x500 dilution of serum was added to each well and incubated for 2 h at 37°C. After discarding the supernatants and washing three times with PBS/Tween, 100 µl of a 5×10^3^ dilution of goat anti-mouse IgG or IgM conjugated to horseradish peroxidase in PBS/Tween was added. After incubation for 1 h at 37°C, the supernatant was discarded, and the plates were washed three times with PBS/Tween. The enzyme-linked Ab bound to the well was revealed by adding 100 µl/well of 1,2-phenylenediamine (0.5 mg/ml) in a 0.1 M citrate/phosphate buffer (pH 5.5) containing 0.003% hydrogen peroxide. The reaction was terminated by the addition of 2 M sulfuric acid (50 µl/well), and the absorbance at 492 nm was read using a micro-ELISA plate reader.

### Immunoblot Analysis of Serum Proteins

The serum samples from the MFG-E8^+/+^ and MFG-E8^−/−^ mice were electrophoresed through a 10% polyacrylamide gel. After electrophoresis, the gel was transblotted onto a nitrocellulose or polyvinylidene difluoride (PVDF) membrane (GE Healthcare), incubated with skim milk for blocking, washed, and then incubated with an HRP-linked secondary Ab. This procedure was followed by the addition of ECL reagents. The bands were visualized using a Cool Saver AE-6955 (ATTO, Tokyo, Japan).

### Preparation of IgM mAbs from MFG-E8^−/−^ mice

Spleen cells from 60-week-old MFG-E8^−/−^ mice were fused with P3/U1 murine myeloma cells and cultured in HAT (hypoxantine/aminopterin/thymidine) selection medium. The culture supernatants of the hybridoma were screened using an ELISA, employing pairs of wells in microtiter plates on which were absorbed calf thymus DNA and ONE-treated BSA as antigens (0.5 µg of protein or DNA per well). After incubation with 100 µl of the hybridoma supernatants, and with intervening washes with Tris-buffered saline, pH 7.8, containing 0.05% Tween 20 (TBS-Tween), the wells were incubated with HRP-conjugated goat anti-mouse IgG, followed by a substrate solution containing 0.5 mg/ml 1,2-phenylenediamine. Hybridoma cells, corresponding to the supernatants that were positive on either DNA or ONE-modified BSA and negative on native BSA, were then cloned by limited dilution. After repeated screenings, two clones showing the most distinctive recognition of DNA and two clones showing the most distinctive recognition of the ONE-modified BSA were obtained.

### Antibody Sequence Analysis

Immunoglobulin variable region genes were cloned and sequenced following amplification by PCR. The total RNA was prepared from 5×10^6^ hybridoma cells by the phenol-guanidine isothiocyanate method (TRIzol Reagent; Life Technologies, Gaithersburg, MD) according to the manufacturer’s protocol. The first-strand cDNA synthesis was performed with RevertAid Reverse Transcriptase (Thermo Scientific) using the manufacturer’s protocol. A 5-µg sample of the total RNA was primed with 10 pmol random primers. Variable region genes were amplified using degenerate sense primers homologous to the mouse heavy and light chain leader sequences and antisense constant primers (Novagen), as previously described [17]. The amplification products were ligated into the pGEM-T Easy Vector (Promega) using standard protocols, and both strands of inserts were sequenced using an automated dye-chain termination DNA sequencer. The Basic Local Alignment Search Tool (BLAST) protocol was used to search the GenBank database to determine the homology with the V regions of other murine Abs that have been sequenced [18].

### Glomerular Immunoglobulin Deposition

For light microscopy, kidney tissues were fixed and embedded in paraffin, and 4 µm sections were stained with H&E. For immunofluorescence, paraffin sections fixed in 4% paraformaldehyde were stained with the Alexa Fluor 488-conjugated antibody against mouse IgG (Molecular Probes, Invitrogen, Carlsbad, CA, USA). Images were digitally captured using a laser scanning confocal microscope and Pascal LSM software (LSM5 Pascal, Zeiss, Jena, Germany).

### Antibody Elution Study

The kidneys were thawed, minced, and homogenized in a high-speed blender for 1 min in 4 volumes of cold PBS, and then repeatedly washed in PBS. The washed homogenates were then incubated with 1 ml glycine-HCl buffer (20 mM, pH 3.0) at room temperature for 10 min, centrifuged at 1,500 rpm, and the pH of the supernatant adjusted to pH 7.4. The eluates were dialyzed overnight against PBS at 4°C. The samples were assessed for anti-DNA and anti-ONE titers.

### Antibody Binding Assay

Jurkat cells (human T cell lymphoma) were cultured in RPMI 1640 medium (Sigma) supplemented with 10% heat-inactivated fetal bovine serum (FBS) (Biowest) (v/v), a 1% penicillin-streptomycin mixed solution (Nacalai Tesque, Kyoto), NaHCO_3_ (2 mg/ml) and glutamine (2 mM). Apoptosis was induced in the Jurkat cells in 6-well plates (5×10^5^ cells/ml; 2 ml/well) by incubation with 10 µM etoposide (Sigma) for 12 h using RPMI 1640 medium containing 10% FBS. Necrosis was induced by a freeze-thawing, alternating between liquid nitrogen and 37°C. Necrotic cell supernatants were separated from necrotic cell pellets by centrifugation at 1,000 rpm at 4°C. The pellets were washed in PBS, centrifuged, and resuspended in 3 ml of fresh cell culture medium. The cells were incubated with 50 µg/ml Abs from the MFG-E8^−/−^ mice or anti-KLH IgM isotype control Ab (MBL) in PBS with 3% BSA for 60 min at 37°C. The cells were washed with ice-cold PBS in triplicate, then incubated with x500 diluted Alexa Fluor 488 goat anti-mouse IgM (μ chain) (Invitrogen) for 15 min on ice. After incubation with the secondary Ab, the cells were incubated with 1 µg/ml PI for 5 min on ice. The fluorescence of 20,000 cells harvested from each well was analyzed by FACS JSAN (Bay Bioscience). The flow cytometric profiles were analyzed using FlowJo software (Tree Star).

The binding of the ONE-specific IgM mAbs to the apoptosis-induced cells was also evaluated using the Annexin-V/PI protocol. The apoptosis-induced cells were incubated with the ONE-specific IgM mAbs from the MFG-E8^−/−^ mice or anti-KLH IgM isotype control Ab (MBL) in PBS with 3% BSA for 30 min at 37°C. The cells were washed with ice-cold PBS in triplicate and then incubated with x500 diluted Alexa Fluor 488 goat anti-mouse IgM (µ chain) (Invitrogen) for 15 min on ice. After incubation with the secondary Abs, the cells were incubated with 2 µg/ml PI and 5 µl of Alexa Fluor® 647 Annexin V (Biolegend) in 50 µl of Annexin V Binding Buffer (MBL) for 15 min at room temperature. The fluorescence of 20,000 cells harvested from each well was analyzed by FACS JSAN (Bay Bioscience). The flow cytometric profiles were analyzed using FlowJo software (Tree Star).

### Annexin-V Competition Assay

Apoptosis was induced in the Jurkat cells (1×10^6^ cells/ml) by incubation with 10 µM etoposide (Sigma) for 15 h, using RPMI 1640 medium containing 10% FBS. The cells were incubated with the IgM mAbs (0, 50, and 100 µg/ml) from the MFG-E8^−/−^ mice or anti-KLH IgM isotype control Ab and 3 µl of Alexa Fluor® 647 Annexin V (Biolegend) in 50 µl of Annexin V Binding Buffer (MBL) for 15 min at room temperature. The fluorescence of 20,000 cells harvested from each well was analyzed by FACS JSAN (Bay Bioscience). The flow cytometric profiles were analyzed using FlowJo software (Tree Star).

### Phagocytosis Assays

THP-1 cells (human acute monocytic leukemia cell line) in RPMI 1640 with 10% FBS were plated in 24-well plates at 2×10^5^ cells/well with 20 nM phorbol myristate acetate (PMA) at 37°C in a 5% CO_2_ atmosphere for 4–5 days. Prior to the phagocytosis assay, the PMA-treated THP-1 cells were labeled with a 2 µM final concentration of CellTrace Far Red (Molecular Probes) for 15 min at 37°C. Afterward, the cells were washed three times with FBS-free RPMI and resuspended in FBS-free RPMI. The Jurkat cells (2×10^6^ cells/ml) were treated with 10 µM etoposide for 15 h at 37°C in RPMI 1640 with 10% FBS and then labeled with 2 µM final concentration of CellTracker Green (Molecular Probes) for 15 min at 37°C. Afterward, the cells were washed three times with FBS-free RPMI and resuspended in FBS-free RPMI. To assess the phagocytosis of the apoptotic cells by the THP-1 cells, 2×10^6^ labeled Jurkat cells were added to THP-1 cell-containing wells in the absence or presence of mAbs (100 µg/well), then incubated in FBS-free RPMI containing 5% mouse sera from Balb/c mice for 3 h at 4°C or 37°C with gentle stirring. The wells were washed five times with ice-cold PBS and fixed with 2% paraformaldehyde. The THP-1 cells in each well were harvested by scraping with a rubber policeman, then washed with PBS. The fluorescence of 20,000 macrophages harvested from each well was analyzed by FACS JSAN (Bay Bioscience). The flow cytometric profiles were analyzed using FlowJo software (Tree Star).

## Results

### Enhanced Production of IgM Antibodies in the MFG-E8^−/−^ Mice

**Figure 2:**
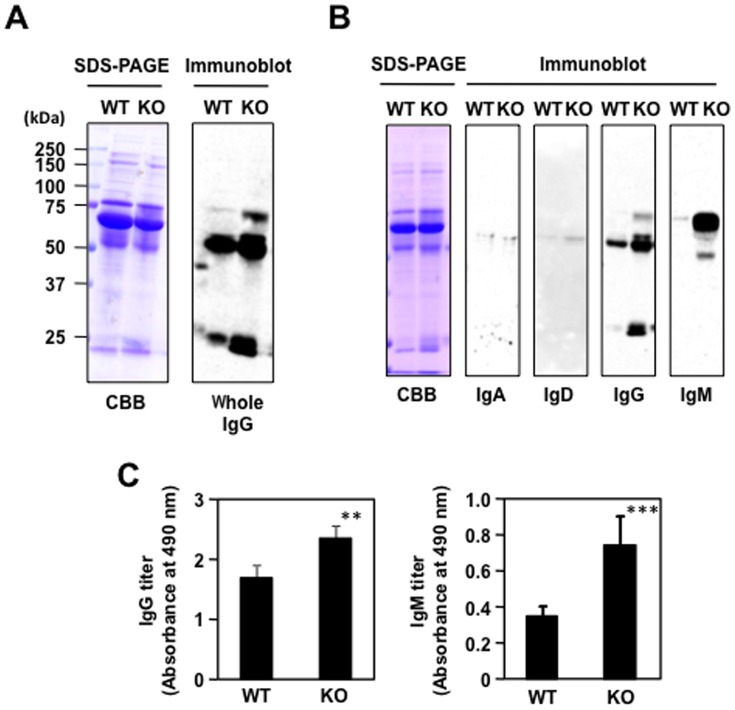
Enhanced production of IgM antibodies in the MFG-E8^−/−^ mice. (**A**) Immunoblot analysis of the MFG-E8^+/+^ (WT) and MFG-E8^−/−^ (KO) mice sera using the anti-mouse whole IgG Ab that cross-reacts not only with IgG, but also with IgM. *Left panel*, SDS-PAGE analysis. *Right panel*, immunoblot analysis. CBB, Coomassie Brilliant Blue. (**B**) Identification of the immunoglobulin class. The MFG-E8^+/+^ and MFG-E8^−/−^ mice sera were analyzed by SDS-PAGE followed by an immunoblot analysis using the antibodies against individual immunoglobulins. (**C**) ELISA analysis of serum IgG and IgM levels in the MFG-E8^−/−^ mice compared to those in the wild-type mice. In panels A and B, the results are representative of at least three experiments with similar results. In panel C, the results represent the means ± SD of three separate experiments performed in duplicate determinations. Asterisks indicate a significant difference between WT and KO groups: **, P<0.01; ***, P<0.001. Differences were analyzed by the unpaired two-tailed Student’s t test.

The MFG-E8^−/−^ mice have been shown to spontaneously produce autoAbs in an age-dependent manner [2]. Indeed, when the MFG-E8^+/+^ and MFG-E8^−/−^ mice sera were analyzed by an immunoblot analysis with the anti-whole IgG Ab, a significant increase in the proteins cross-reacted with the Ab was observed in the MFG-E8^−/−^ mice (**Figure 2A**). Based on their molecular masses, they were suggested to be IgG and IgM Abs. To further characterize the Ab isotype patterns, the MFG-E8^+/+^ and MFG-E8^−/−^ mice sera were analyzed by SDS-PAGE and an immunoblot analysis. Among the immunoglobulins (IgA, IgD, IgG, and IgM) tested, both IgA and IgD were nearly undetectable in both groups, whereas IgG was detectable in the MFG-E8^+/+^ mice and its level also increased in the MFG-E8^−/−^ mice. The most drastic change was observed in the IgM. The protein was barely detected in the MFG-E8^+/+^ sera whereas it appeared to be highly produced in the MFG-E8^−/−^ mice (**Figure 2B**). The ELISA analysis also showed a significant elevation in the serum IgM levels of the MFG-E8^−/−^ mice compared to those in the wild-type mice (**Figure 2C**). Thus, it appeared that the production of immunoglobulins, IgM in particular, is highly upregulated in the MFG-E8-deficient mice.

### Recognition of Oxidation-specific Epitopes by the MFG-E8^−/−^ Mice Sera

**Figure 3:**
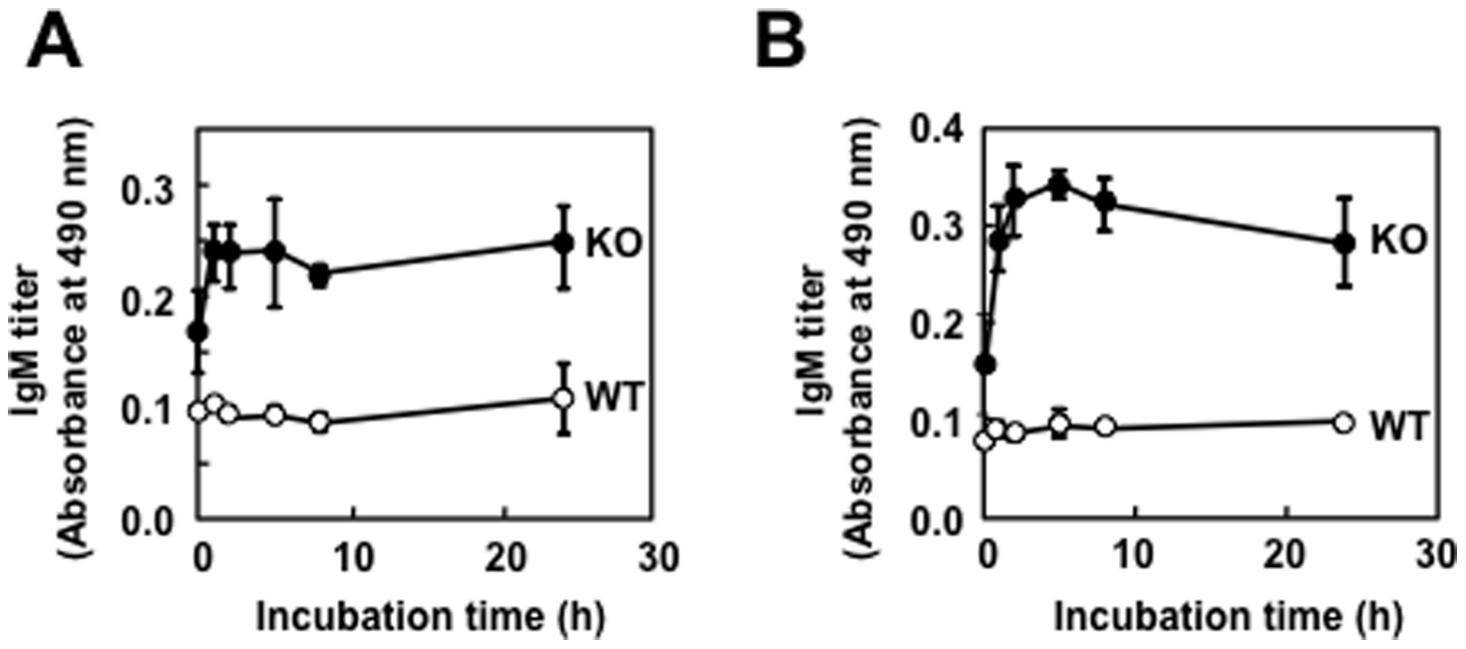
Recognition of lipid peroxidation-modified proteins by the MFG-E8^−/−^ mice sera. BSA was incubated with a polyunsaturated fatty acid (linoleic acid or arachidonic acid) in the presence of the Fe^2+^/ascorbate free radical-generating system. Cross-reactivity of the modified proteins with the sera from MFG-E8^+/+^ and MFG-E8^−/−^ mice was examined by ELISA. (**A**) Formation of lipid peroxidation-derived epitopes upon the reaction of BSA with peroxidized linoleic acid. *Symbol*s: *closed circle*, MFG-E8^−/−^ mice; *open circle*, MFG-E8^+/+^ mice. (**B**) Formation of lipid peroxidation-derived epitopes upon the reaction of BSA with peroxidized arachidonic acid. *Symbols*: *closed circle*, MFG-E8^−/−^ mice; *open circle*, MFG-E8^+/+^. In panels A and B, the results represent the means ± SD of three separate experiments performed in duplicate determinations.

**Figure 4:**
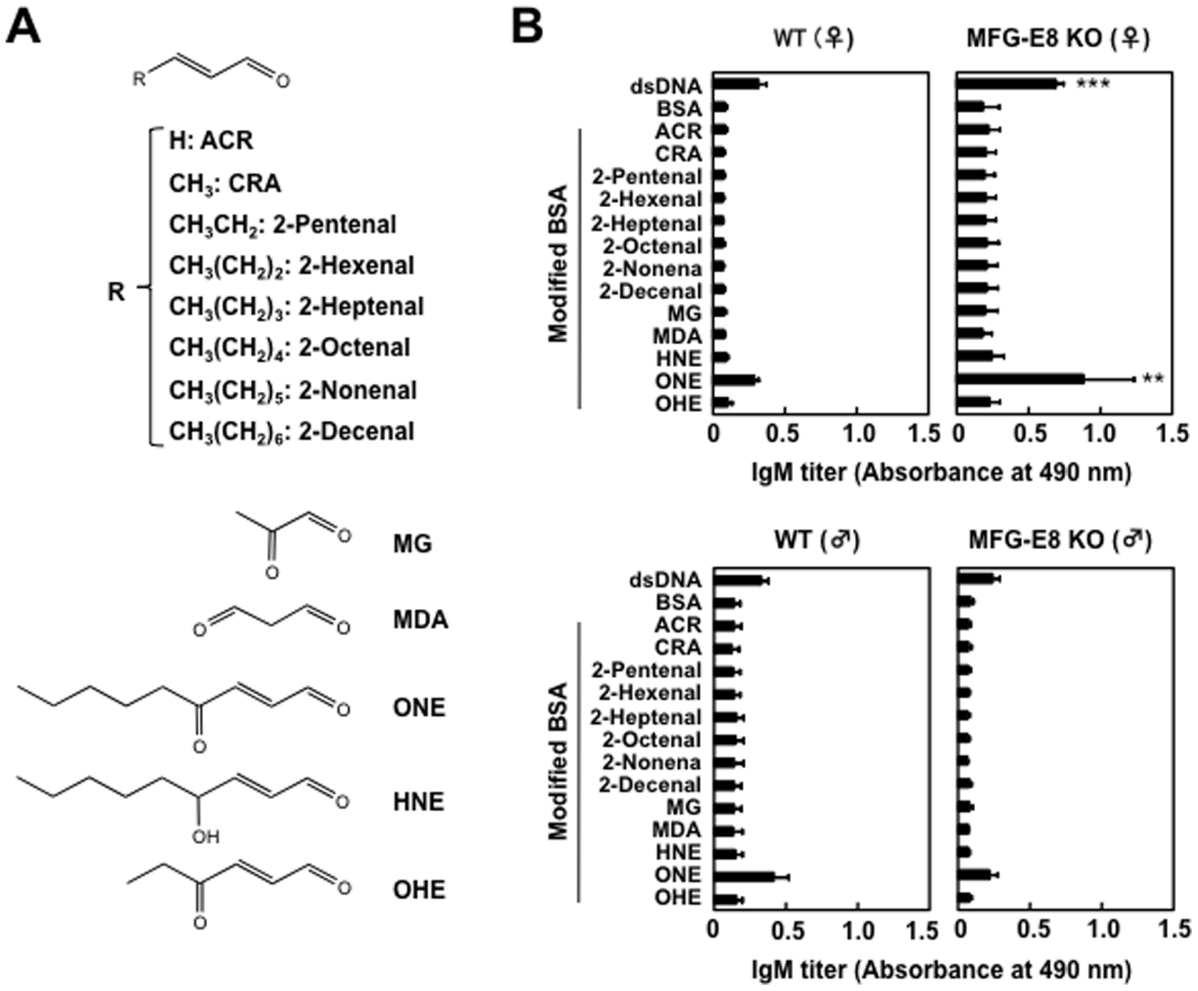
Identification of ONE as a source of lipid peroxidation-derived epitopes. (**A**) Structures of lipid peroxidation-derived aldehydes used in this study. (**B**) Cross-reactivity of the MFG-E8^+/+^ and MFG-E8^−/−^ mice sera with the aldehyde-modified proteins. *Left panels*: MFG-E8^+/+^mice (*upper*: female, *lower*: male). *Right panels*: MFG-E8^−/−^ mice (*upper*: female, *lower*: male). The IgM titer of the mice sera was determined by a direct antigen ELISA using DNA and native and aldehyde-modified BSA as the absorbed antigens. In panels A and B, the results represent the means ± SD of three separate experiments performed in duplicate determinations. Asterisks indicate a significant difference between BSA and DNA groups and between BSA and ONE-modified BSA groups: **, P<0.01; ***, P<0.001. Differences were analyzed by the unpaired two-tailed Student’s t test.

The exact reasons for the relatively high incidences of basal IgM antibody levels in comparison to the wild-type mice are still unknown. However, based on the previous studies showing that lipid peroxidation-damaged molecular complexes called “oxidation-specific epitopes” are an important target of innate natural IgM antibodies [19], [20], we sought to determine whether the MFG-E8^−/−^ mice sera display binding to the oxidation-specific epitopes. To this end, BSA was incubated with a polyunsaturated fatty acid (arachidonic acid or linoleic acid) in the presence of the Fe^2+^/ascorbate free radical-generating system and evaluated the IgM titers using an ELISA with the MFG-E8^−/−^ mice sera. Along with the progress in the peroxidation of linoleate, the modified protein showed significant IgM titers to the MFG-E8^−/−^ mice sera, whereas no IgM titers to the sera from the MFG-E8^+/+^ mice were observed (**Figure 3A**). A similar increase in the IgM titers to the MFG-E8^−/−^ mice sera was obtained when the protein was incubated with peroxidized arachidonate (**Figure 3B**). Thus, the peroxidation of these polyunsaturated fatty acids is likely to generate product(s), which may have the ability to cause formation of lipid peroxidation-derived epitopes that cross-react with the MFG-E8^−/−^ mice sera. To identify the source of the lipid peroxidation-derived epitopes recognized by the MFG-E8^−/−^ mice sera, we further assessed the specific IgM titers to the modified proteins with unsaturated aldehydes of chain lengths varying from three to nine carbons. Among the modified proteins tested, the IgM responses to the ONE modification were consistently found to be the most robust in the female MFG-E8^−/−^ mice, and the titers were almost comparable to those of the native DNA (**Figure 4A, B**). These data suggest the presence of IgM Abs in MFG-E8^−/−^ mice that specifically cross-react with the ONE-modified proteins.

### Evidence for the Presence of ONE-specific IgM Abs in the MFG-E8^−/−^ Mice

**Figure 5:**
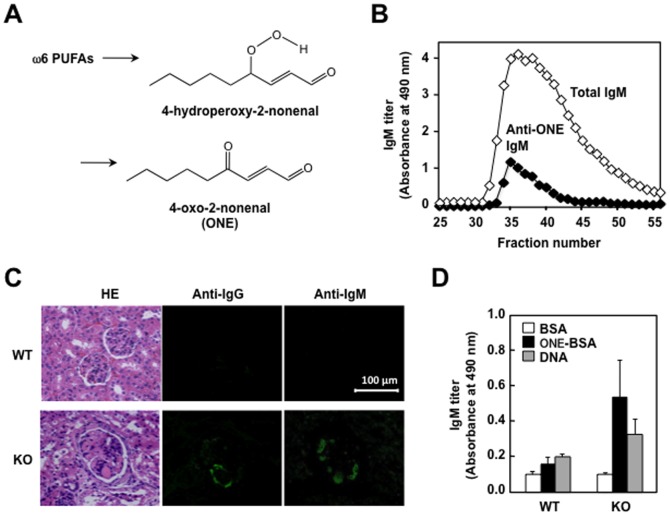
Presence of ONE-specific IgM antibodies in the MFG-E8^−/−^ mice. (**A**) Formation of ONE via 4-hydroperoxy-2-nonenal during the peroxidation of ω6 polyunsaturated fatty acids. (**B**) Separation of MFG-E8^+/+^mice sera by gel filtration. The serum was eluted with PBS at the flow rate of 0.5 ml/min at room temperature with monitoring of the absorbance at 280 nm. The column system was composed of Hi Prep 16/60 Sephacryl S-300. The total IgM was determined by a direct antigen ELISA using the anti-IgM antibodies. The anti-ONE IgM titers were determined by a direct antigen ELISA using the ONE-modified BSA as the absorbed antigens. (**C**) Glomerular immunoglobulin deposition in 60-week-old female MFG-E8^+/+^ (WT) and MFG-E8^−/−^ (KO) mice. Kidney sections were stained with Alexa Fluor 488-conjugated antibody against mouse IgG or IgM. *HE*: Hematoxylin and Eosin. (**D**) Cross-reactivity of antibodies eluted from the kidneys of MFG-E8^+/+^ (WT) and MFG-E8^−/−^ (KO) mice. The IgM titer of the antibodies was determined by a direct antigen ELISA using native BSA, ONE-treated BSA, and DNA as the absorbed antigens. In panels B and C, the results are representative of three separate experiments with similar results. In panel D, the results represent the means ± SD of three separate experiments performed in duplicate determinations. There were no statistically significant differences between WT and KO groups.

ONE is one of the major lipid peroxidation products formed by the free radical-initiated degradation of ω6 polyunsaturated fatty acids, such as linoleic acid and arachidonic acid (**Figure 5A**), and is known to be a potent genotoxin, which covalently reacts with protein and DNA [21], [22]. To obtain evidence for the presence of ONE-specific IgM Abs in the MFG-E8^−/−^ mice, the serum from the mice was fractionated using a gel-filtration column, and the total serum IgM and anti-ONE IgM titers were assessed by ELISA. As shown in **Figure 5B**, the anti-ONE IgM indeed co-eluted with the total IgM. On the other hand, because serum autoAbs are directly involved in the formation of immune complexes, we also examined the deposition of IgG and IgM in the glomeruli of the MFG-E8^+/+^ and MFG-E8^−/−^ mice. The MFG-E8^−/−^ mice indeed had a deposit of these immunoglobulins, mostly localized in the glomeruli, whereas no glomerular immunoglobulin deposition was present in the wild-type mice (**Figure 5C**). Furthermore, we also assessed the specificity of the Ab that was deposited in the glomeruli of the MFG-E8^−/−^ mice and observed that the titers of the anti-ONE IgM were higher in the MFG-E8^−/−^ mice than in the MFG-E8^+/+^ mice (**Figure 5D**).

### Characterization of ONE-specific IgM mAbs

**Figure 6:**
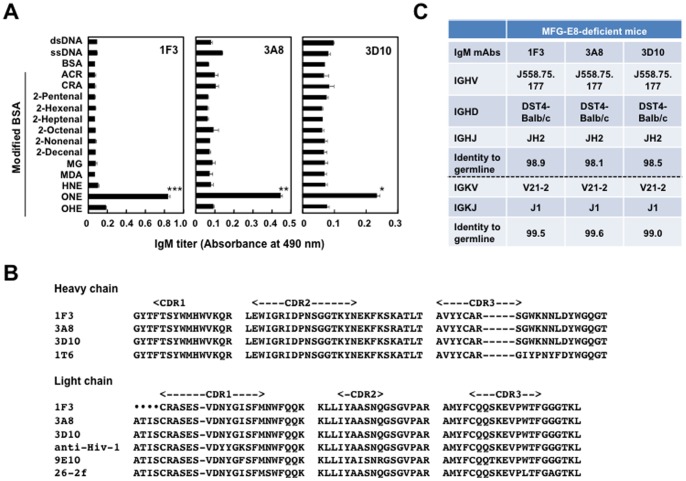
Characterization of ONE-specific IgM mAbs. (**A**) Cross-reactivity of the ONE-specific IgM mAbs established from the MFG-E8^−/−^ mice. The IgM titer of the mAbs was determined by a direct antigen ELISA using dsDNA and native and aldehyde-modified BSA as the absorbed antigens. The results represent the means ± SD of three separate experiments performed in duplicate determinations. Asterisks indicate a significant difference between BSA and ONE-modified BSA groups: *, P<0.05; **, P<0.01; ***, P<0.001. Differences were analyzed by the unpaired two-tailed Student’s t test. (**B**) Sequence alignment of the hypervariable regions of ONE-specific IgM mAbs prepared from female MFG-E8^−/−^ mice. CDR1, CDR2, and CDR3 represent the Kabat definition complementarity-determining regions (CDRs). Hyphens (−) indicate sequence gaps, and the dots (•) indicate the sequence not determined. Sequences were aligned using the program CLUSTALW and were manually modified. Accession numbers for the sequences are as follows. Gen Bank: 9E10, CAN87019. Protein data bank: 26-2f, 1H0D; anti-Hiv-1, 1MF2. (**C**) V region gene use of ONE-specific IgM mAbs. All sequencing analyses were performed at least three times.

Based on the finding that the IgM Abs against the ONE-modified proteins are present in the MFG-E8^−/−^ mice sera, we sought to isolate the hybridoma clones, producing the Abs specific for the ONE-modified proteins and/or DNA, from the MFG-E8^−/−^ mice. Spleen cells from female MFG-E8^−/−^ mice at 60 weeks of age were fused with P3/U1 murine myeloma cells and, after screening based on specific binding to the ONE-modified BSA and DNA, we established three hybridoma clones, 1F3, 3A8, and 3D10, producing the mAbs specific for the ONE-modified proteins and two hybridoma clones, 3B6 and 4E11, producing the mAbs specific for DNA (**Figure S1**). It was revealed that ONE, among the tested lipid peroxidation-derived reactive aldehydes, was the only source of the immunoreactive structures recognized by the anti-ONE IgM Abs (**Figure 6A**).

The V_H_ and V_L_ nucleotide sequences and the corresponding gene families were determined for the anti-ONE IgM mAbs 1F3, 3A8, and 3D10 (**Figure 6B**). The identity of their V region genes was determined by searching the GenBank database for homologies to known V region genes using the BLAST protocol [18]. All of the anti-ONE IgM mAbs arising from MFG-E8^−/−^ mice showed a 98% nucleotide homology with the J558 subfamily gene (**Figure 6C**), suggesting that these IgM mAbs cloned from these mice might represent an expansion of the population of natural Abs ubiquitously expressed in healthy individuals. A homology search revealed that the V_H_ domains of these mAbs were almost identical (98% homology). A sequence identity of greater than 90% for the V_H_ domains was shared with the anti-4-hydroxy-3-nitrophenylacetyl mAb 1T6 [23]; however, no sequence similarity with the anti-DNA autoAbs was observed. The V_L_ domain of the anti-ONE IgM Abs was completely identical, showing the sequence identity of 96∼98% with the anti-human c-myc [24], anti-angiogenin [25], and anti-HIV-1 protease antibodies [26].

We also characterized the sequence of the anti-DNA IgM mAbs, 3B6 and 4E11 (**Figure S2**). To confirm that these antibodies represent the anti-DNA Abs, the V region sequences were compared. A homology search revealed that the sequence identity of only 82% for the V_H_ domain of 3B6 was shared with 4E11. However, the V_H_ gene of 3B6 revealed a sequence similarity (87–89%) with the anti-DNA Abs (1DD10, 1DC10, 1DC7, and C12B5H) and with the autoAbs (BL7-4, H121, and H290) [27], [28]. In addition, the V_H_ gene of 4E11 also revealed a sequence similarity (87–89% identity) with the anti-DNA Abs, anti-DNA/SmRNP H161, and autoAbs H175 and H295 [29]. The V_L_ gene of 3B6 revealed a sequence similarity with the anti-Mn 423 (95% identity) [30] and anti-DNA DNA-1 (81% identity) [31]. The sequence identity of 97% with an anti-DNA was observed for the V_L_ domain of 4E11 [32].

### Recognition of Apoptotic/Necrotic Cells by the ONE-specific IgM mAbs

**Figure 7:**
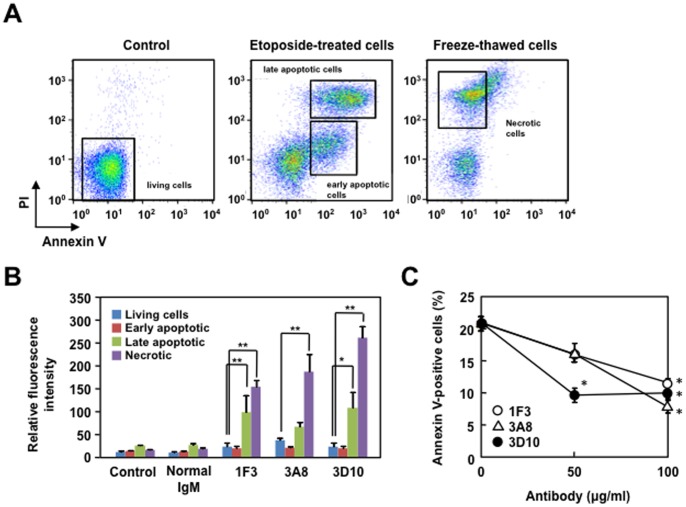
The binding of the ONE-specific IgM mAbs to the apoptosis-induced cells evaluated using Annexin-V/PI. (**A**) Apoptosis-induced Jurkat cells were incubated with monoclonal antibodies and gated into populations according to the Annexin V and PI intensity; early apoptotic cells (single positive in Annexin-V but not PI) and late apoptotic and necrotic cells (double positive in Annexin-V/PI staining). Necrosis in Jurkat cells was induced by a freeze-thawing. (**B**) Bar graph representing relative fluorescence intensity of antibody binding to living cells, early apoptotic cells, late apoptotic cells, and necrotic cells. The non-immune control murine IgM and the ONE-specific IgM mAbs, 1F3, 3A8, and 3D10, isolated from the MFG-E8^−/−^ mice, were used. The means were tested for statistical significance by using Tukey’s HSD test, assuming equal variances. Statistically significant differences between the relative fluorescence intensity of living cells are indicated by asterisks (*, P<0.05; **, P<0.01). (**C**) Inhibition of Annexin-V binding to apoptotic Jurkat cells by the ONE-specific IgM mAbs. The apoptosis-induced Jurkat cells were incubated with the ONE-specific IgM mAbs (0, 50, and 100 µg/ml) and Annexin-V and the binding of Annexin V to the cells was analyzed by flow cytometry.

One of the mechanisms proposed to explain the pathogenicity of the autoAbs relies on their ability to either remain on the cell surface and participate in the complement-mediated cytotoxicity, or can be internalized to traverse the cytoplasm and localize in the nucleus [33]. It has also been demonstrated that the natural IgM Abs against the oxidation-specific epitopes bind to apoptotic cells, but not to normal cells [34]. To test this idea, the ability of the ONE-specific IgM mAbs to bind to the apoptosis-induced Jurkat cells was examined. The binding of the ONE-specific IgM mAbs to apoptotic cells was evaluated using Annexin-V, which specifically binds to phosphatidylserine. As shown in **Figure 7** (panels A–C), the mAbs significantly recognized the Annexin-V/PI positive cells whereas the recognition of the cells by Annexin-V was partially inhibited by the mAbs. In addition, the mAbs recognized necrotic cells induced by freeze-thawing. These data suggest that the cells recognized by the mAbs include both late apoptotic and necrotic cells.

### The ONE-specific IgM mAbs Enhance Phagocytosis of Apoptotic/Necrotic Cells by THP-1 Cells

**Figure 8:**
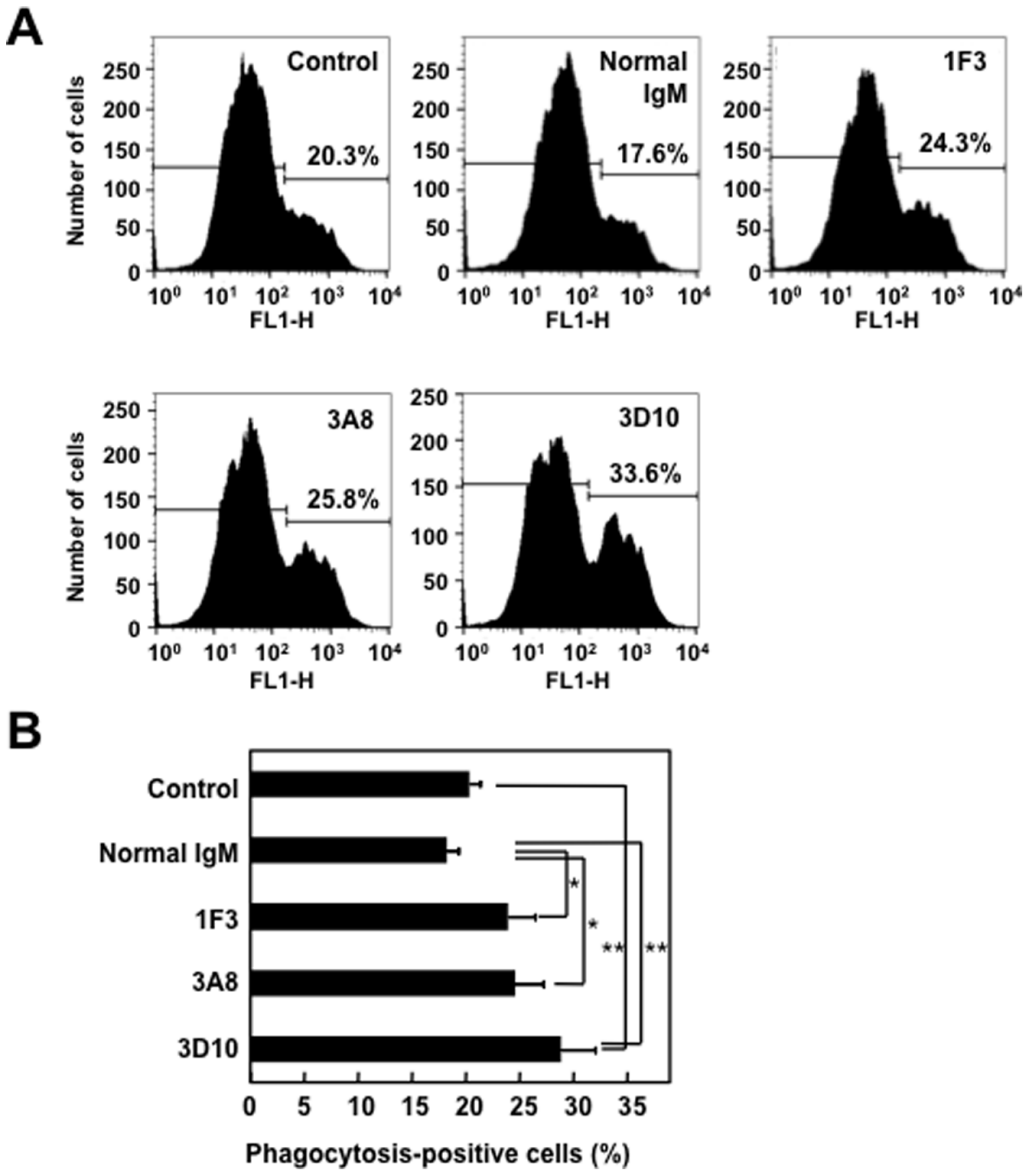
The ONE-specific IgMs promote the uptake of apoptotic cells by THP-1 monocytes. CellTracker Green-labeled Jurkat cells were induced to undergo late apoptotsis, followed by incubation with CellTrace Far Red-lableled THP-1 cells. After washing and fixation, the THP-1 cells were harvested by scraping with a rubber policeman. The percentage of double-positive macrophages was analyzed by flow cytometry. (**A**) Flow cytometric analysis of THP-1 cells engulfing apoptotic Jurkat cells in the presence and absence of IgM. The results are representative of three separate experiments with similar results. (**B**) Percentages of phagocytosis-positive THP-1 cells engulfing apoptotic Jurkat cells in the presence and absence of IgM. Data represented the mean ± SD of triplicate experiments. The means were tested for statistical significance by using Tukey’s HSD test, assuming equal variances. Statistically significant differences between the control and normal IgM values are indicated by asterisks (*, P<0.05; **, P<0.01).

The natural IgM Abs that recognize apoptotic cells have been shown to enhance the phagocytic clearance of dead/dying cells and to suppress the innate immune signaling pathways [35]. Hence, to test the ability of the IgM Abs to enhance the uptake of apoptotic cells, we repeated the phagocytosis experiment in the absence and presence of the ONE-specific IgM mAbs. The representative data are shown in **Figure 8A**. In the absence of added mAb, 20.1% of the THP-1 cells took up the Jurkat cells. 1F3 enhanced this to 23.7%, 3A8 to 24.4%, and 3D10 to 28.6%. **Figure 8B** summarizes the results of multiple experiments demonstrating the ability of the mAbs to enhance uptake. These findings and the observations that (i) the nonimmune murine IgM showed an inhibitory effect on the uptake and (ii) no phagocytosis occurred at 4°C (data not shown) suggest that the ONE-specific IgM mAbs indeed enhanced phagocytosis of dead/dying cells.

## Discussion

The IgM Abs are produced at tightly regulated levels in the complete absence of external antigenic stimulation. They provide immediate, early and broad protection against pathogens, making them a crucial non-redundant component of the humoral immune system [19], [20]. They have also been shown to play an important function in the host response to the consequences of oxidative stress during oxidative events that occur when cells undergo apoptosis [35]. These Abs are mainly produced by a subset of long-lived, self-replenishing B cells termed B-1 cells. It has been shown that B-1 cells can also respond to certain antigenic stimuli, e.g., 1, 3-dextran, when presented on an appropriate carrier or in an appropriate immunization vehicle [36]. In the current study, we observed that the MFG-E8 deficiency was accompanied by the increased production of immunoglobulins. Of interest, the MFG-E8^−/−^ mice showed a prominent increase in the IgM isotype compared to the modest increase in the IgG isotype. The mechanism(s) for elevation of the IgM Abs in the MFG-E8^−/−^ mice is presently unknown. However, it may be likely that the IgM Abs found associated with the impairment of phagocytic clearance of apoptotic cells represents an expansion of the population of these Abs ubiquitously expressed in normal healthy mice or, alternatively, represent the products of an antigen-selected B lymphocyte population. Indeed, the established B-1 cell clones can be expanded by antigen exposure, leading to the increased IgM levels in the plasma [37].

**Figure 9:**
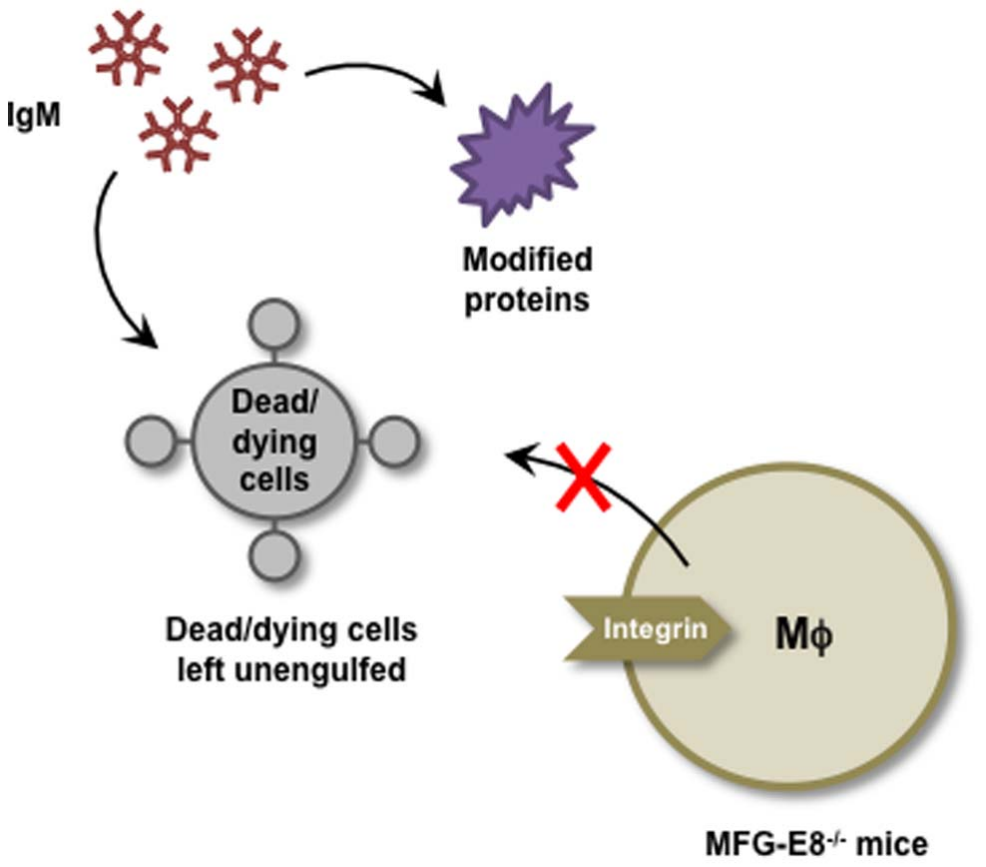
Schematic representation of the mechanism by which MFG-E8 deficiency is associated with the enhanced production of IgM Abs that specifically recognize the ONE-modified proteins and phosphatidylserine exposed on the outer leaflet of the apoptotic cell membranes. Note that the possibility of the presence of ONE-derived epitopes in apoptotic cells cannot be eliminated.

Chou et al. [38] proposed that the oxidation-derived epitopes generated on self-antigens are important immunodominant targets of natural IgM Abs. It has also been demonstrated that the IgM Abs specifically bind to lipid peroxidation-derived products, such as oxidized phospholipids and malondialdehyde, which form neo-self determinants on dying cells and oxidized low-density lipoproteins [39]. We found in this study that the MFG-E8^−/−^ mice sera cross-reacted with the oxidation-specific epitopes generated upon incubation of the serum albumin with peroxidized lipids. In addition, among the modified proteins with several unsaturated aldehydes of chain lengths varying from three to nine carbons, the MFG-E8^−/−^ mice sera exclusively cross-reacted with the protein-bound ONE. The apparent high prevalence of the ONE-modified proteins as targets of the sera likely reflects the ubiquitous presence of these modified proteins consequent to oxidative events. Moreover, based on the finding that the IgM Abs against ONE-modified proteins are present in MFG-E8^−/−^ mice sera, we sought to isolate the hybridoma clones, producing the Abs specific for the ONE-modified proteins, and established three ONE-specific IgM mAbs, 1F3, 3A8, and 3D10. We also established the anti-DNA IgM Abs, 4E11 and 3B6, from the mice, indicating the existence of at least two distinct populations of B cell clones in the MFG-E8^−/−^ mice; one clone produces the Abs specific to the ONE-modified proteins; the other clone produces the Abs specific to the native DNA. Thus, the deficiency of MFG-E8 was associated with the enhanced production of IgM Abs that specifically recognize the ONE-modified proteins or DNA (**Figure 9**).

In our previous study, we revealed a similar spontaneous age-dependent increase in the immune response to the ONE-modified proteins in MRL-*lpr* mice, which carry a defective apoptosis-related *Fas* gene and develop a spontaneous lupus-like disease as they age [14]. Intriguingly, a subset of the anti-DNA IgG mAbs established from the MRL-*lpr* mice, showing a recognition specificity toward DNA, cross-reacted with the ONE-modified proteins. The impaired uptake of apoptotic cells may therefore be commonly associated with the increased production of IgG and/or IgM autoAbs against the modified proteins. However, it is also notable that the ONE-specific mAbs established from the MFG-E8^−/−^ mice are different from those established from the MRL-*lpr* mice in several aspects. (i) The isotype of mAbs is different. The mAbs obtained from the MRL-*lpr* mice are IgGs whereas the mAbs from the MFG-E8-deficient mice are IgMs. This may be critical because IgG is generally believed to be involved in adaptive immune response whereas IgM is in innate immunity. (ii) The ONE-specific IgGs from the MRL-lpr mice show recognition specificity toward DNA, whereas the ONE-specific IgMs from MFG-E8^−/−^ mice hardly cross-react with DNA. (iii) The ONE-specific IgGs from the MRL-*lpr* mice bind and internalize into living cells, whereas the ONE-specific IgMs from MFG-E8^−/−^ mice bind late apoptotic/necrotic cells. These differences may be due to the production of the Abs from distinct B cell subsets. The ONE-specific IgM Abs may represent the product of the B-1 cells ubiquitously expressed in normal healthy mice, whereas the development of the ONE-specific IgGs may occur by a common mechanism in which DNA or a protein-bound ligand stimulates B-2 cells to differentiate in a receptor-mediated response.

ONE is one of the most reactive aldehydic by-products of lipid peroxidation. This highly reactive aldehyde could modify a wide range of substrates, including proteins, lipids, and even DNA, generating ONE neoepitopes in a variety of pathophysiological events, such as ischemia and reperfusion, diabetes, and atherosclerosis, as well as inflammatory events in the brain. Upon reaction with protein, ONE covalently modifies the arginine, cysteine, histidine, and lysine residues [40]. The predominant initial reaction appears to involve the Michael addition to the central ONE double bond, more at C3 than at C2, to produce substituted 4-oxononanals. These adducts are relatively unstable and could be further converted to stable products, which include dihydrofuran, dihydropyrrole, and isomeric 4-ketoamide derivatives originating from the reaction of ONE with lysine [41] and a substituted imidazole derivative with arginine [42]. ONE also forms furan derivatives upon its reaction with cysteine and histidine derivatives [43]–[45]. Based on the reactivity of ONE toward proteins, the ONE-specific mAbs are likely to recognize a specific ONE-amino acid adduct. However, the exact nature of the epitope structure remains unknown.

The reactivity of ONE may be closely associated with its apoptotic function. Indeed, West et al. [46] compared the apoptotic responses induced by a series of toxic α,β-unsaturated aldehydes, including ONE, in a human colorectal cancer cell line and demonstrated that the apoptotic response induced by ONE and other related aldehydes involves the activation of caspases, proteolysis of downstream caspase targets, and nucleosomal DNA fragmentation. ONE was also shown to induce apoptotic cell death in a wide variety of cell types, partly by modulating the intracellular signaling pathways [47], [48]. Shibata et al. [49] showed that the p53 signaling pathway is involved in the neuronal cell death induced by ONE. More strikingly, the ONE-induced activation of the p53 pathway was found to mediate the gene expression of Fas, suggesting the involvement of the Fas/FasL signaling pathway, which is known to be one of the downstream mediators in the p53-dependent apoptotic pathway. Thus, it may be likely that these pro-apoptotic functions of ONE are involved, at least in part, in the high prevalence of the ONE-specific Abs. An experiment to investigate the role of ONE as a source of autoantigens is currently underway.

The clearance of dying cells is one of the most essential responsibilities of the immune system, which is required to prevent uncontrolled inflammation and autoimmunity. The natural IgM Abs that recognize apoptotic cells have been shown to enhance the phagocytic clearance of dead and dying cells and to suppress the innate immune signaling pathways. It has been suggested that the binding of IgM to apoptotic cells leads to the formation of complexes with the recruitment of the early complement factors, such as C1q, which serve as bridging molecules that trigger specialized phagocyte functions for engulfment and clearance of apoptotic cells, by a pathway that does not require downstream activation of the complement cascade [50], [51]. Chang et al. [52] previously demonstrated that the IgM Abs against oxidation-specific epitopes, such as oxidized phospholipids and malondialdehyde-modified proteins, showed specific binding to the surface of apoptotic cells, but not to the surface of normal cells. They have also shown that the Abs inhibited the macrophage uptake of apoptotic cells, whereas the control IgM did not. Based on these data, they formed a hypothesis that oxidized lipids and/or oxidized lipid-protein adducts on the surface of apoptotic cells, presumably derived from the membrane peroxidation that occurs during apoptosis, are ligands for the phagocytosis of apoptotic cells. In this study, we observed that the natural IgM Abs against the ONE-modified proteins established from the MFG-E8^−/−^ mice could bind the late apoptotic/necrotic cells (**Figures 7**. In addition, we also observed that the ONE-specific IgM mAbs enhanced the phagocytosis of apoptotic cells (**Figure 8**). These data suggest that the MFG-E8 deficiency might be associated with the enhanced production of IgM Abs that recognize phosphatidylserine exposed on the outer leaflet of the dead/dying cell membranes (**Figure 9**). Obviously, further study will be needed to define how these IgM Abs recognize the structurally unrelated molecules, including the ONE-modified proteins and phosphatidylserine.

The IgM autoAbs have been demonstrated to be present at significantly higher levels in the SLE patients with a lower disease activity and with less severe organ damage [35]. Witte [53] proposed a possible mechanism of protection by the IgM Abs, in which the Abs may improve clearance of pathogenic immune complexes containing IgG. Others have suggested that the IgM Abs remove cellular debris and therefore may prevent the formation of IgG Abs [54]. Indeed, in the absence of secreted IgM, normal mice spontaneously develop the autoreactive IgG specific for DNA [55]. It has also been suggested that the IgM Abs play a protective role against the development of glomerulonephritis [56]. In addition, in the SLE-prone MRL-*lpr* mice, the absence of secreted IgM accelerates the development of IgG autoAbs and glomerulonephritis, and the mice succumb to the disease at an earlier age. Thus, the secreted IgM can suppress the development of IgG autoAbs and autoimmune disease under physiological conditions. Because autoantigenic epitopes would be generated even in healthy individuals, these IgM function may play a vital role in maintaining homeostasis. Our future experiment will test whether the ONE-specific IgMs show protective effect on the phenotype of autoimmune strains.

In summary, we observed that the MFG-E8 deficiency was accompanied by the increased production of immunoglobulins. Of interest, the MFG-E8^−/−^ mice showed a prominent increase in the IgM isotype compared to the modest increase in the IgG isotype. In addition, the IgM antibodies established from the MFG-E8-deficient mice recognized lipid peroxidation-modified proteins, the ONE-modified proteins in particular, as the oxidation-specific epitope. Furthermore, we demonstrated that the oxidation-specific mAbs, originally identified by their ability to bind to the ONE-modified proteins, could bind the apoptotic cells and enhance the phagocytosis. These data suggest that the deficiency of MFG-E8 leads to the enhanced production of IgMs that may mediate the clearance of multiple damaged-associated molecules, including lipid peroxidation-modified proteins and apoptotic cells (**Figure 9**).

## Supporting Information

Figure S1 **Establishment of the ONE- and DNA-specific IgM mAbs from MFG-E8^−/−^ mice.** The IgM titer of the mAbs was determined by a direct antigen ELISA using dsDNA and native and ONE-modified BSA as the absorbed antigens. The results represent the means ± SD of three separate experiments performed in duplicate determinations.
(TIF)

Figure S2 **Sequence alignment of the hypervariable regions of anti-DNA IgM mAbs prepared from female MFG-E8^−/−^ mice.** CDR1, CDR2, and CDR3 represent the Kabat definition complementarity-determining regions (CDRs). Hyphens (−) indicate sequence gaps, and the dots (•) indicate the sequence not determined. Sequences were aligned by using the program CLUSTALW and were manually modified. Accession numbers for the sequences are as follows. Gen Bank: 1DD10 (anti-DNA, unpublished), AAS00743; 1DC7 (anti-DNA, unpublished), AAS00741; C12B5H (anti-DNA, unpublished), AAB53405; 1DC10 (anti-DNA, unpublished), AAS00737; H290 (autoantibody), ACB47989; H121 (autoantibody), ACB47958; BL7-4 (autoantibody), AAC63397; H175 (autoantibody), ACB47970; H295 (autoantibody), ACB47991; H161 (anti-ssDNA/SmRNP), AAB25308; 1ED3 (anti-DNA), AAS00776; 1EA8 (anti-DNA), AAS00764. Protein data bank: anti-Mn423, 2V17; DNA-1, 1I8M; anti-ssDNA, 1CBV. All sequencing analyses were performed at least three times.
(TIF)
